# Co-Therapy of Pegylated G-CSF and Ghrelin for Enhancing Survival After Exposure to Lethal Radiation

**DOI:** 10.3389/fphar.2021.628018

**Published:** 2021-02-02

**Authors:** Juliann G. Kiang, Min Zhai, Bin Lin, Joan T. Smith, Marsha N. Anderson, Suping Jiang

**Affiliations:** ^1^ Radiation Combined Injury Program, Armed Forces Radiobiology Research Institute, Bethesda, MD, United States; ^2^ Department of Pharmacology and Molecular Therapeutics, Uniformed Services University of the Health Sciences, Bethesda, MD, United States; ^3^ Department of Medicine, Uniformed Services University of the Health Sciences, Bethesda, MD, United States

**Keywords:** radiation, survival, G-CSF, ghrelin, enhancement, bone marrow, GI

## Abstract

Exposure to ionizing radiation (radiation injury, RI) in nuclear-related episode is evident to be life-threatening. RI occurs at levels of organs, tissues, cytosols, or nucleus. Their mechanisms are still not fully understood. FDA approves pegylated granulocyte colony-stimulating factor (Neulasta™, Peg-G-CSF) for acute hematopoietic syndrome and has been shown to save lives after lethal RI. We aimed to test whether Ghrelin enhanced Peg-G-CSF’s efficacy to save more lives after lethal RI. B6D2F1/J female mice were used for the study. They received 9.5 Gy (LD50/30 at 0.4 Gy/min) emitted from the ^60^Co-γ-photon radiation facility. Peg-G-CSF was injected subcutaneously at 1 mg/kg once on days 1, 8, and 15 after irradiation. Ghrelin contains 28 amino acid and is a hunger peptide that has been shown to stimulate food intake, promote intestinal epithelial cell proliferation, elevates immunity, inhibits brain hemorrhage, and increases stress-coping. Ghrelin was injected subcutaneously at 113 μg/kg once on days 1, 2, and 3 after irradiation. Survival, body weight, water consumption, hematology, spleen weight, splenocytes, bone marrow cells, and histology of bone marrow and ileum were performed. We observed that radiation resulted in 30-days survival by 30%. RI decreased their body weights and water consumption volumes. On the 30th day post-RI, platelets and WBCs such as basophils, eosinophils, monocytes, lymphocytes, neutrophils and leukocytes were still significantly decreased in surviving mice. Likewise, their RBC, hemoglobin, hematocrit, and splenocytes remained low; splenomegaly was found in these mice. Bone marrow in surviving RI animals maintained low cellularity with high counts of fat cells and low counts of megakaryocytes. Meanwhile, ileum histology displayed injury. However, mice co-treated with both drugs 24 h after RI resulted in 30-days survival by 45% above the vehicle group. Additionally, the body-weight loss was mitigated, the acute radiation syndrome was reduced. This co-therapy significantly increased neutrophils, eosinophils, leukocytes, and platelets in circulation, inhibited splenomegaly, and increased bone marrow cells. Histopathological analysis showed significant improvement on bone marrow cellularity and ileum morphology. In conclusion, the results provide a proof of concept and suggest that the co-therapy of Peg-G-CSF and Ghrelin is efficacious to ameliorate RI.

## Introduction

Detonation of nuclear weapons, radiation dispersal devices, or radiation-power plants and equipment will result in ionizing radiation emission so as to cause injuries, namely, radiation injury (RI) or sometimes followed by thermal energy exposure or blast trauma. *In vivo* (Kiang et al., 2012) and *in vitro* (Fukumoto and Kiang, 2011) studies have demonstrated that RI induces pathophysiological responses, including elevation of DNA double-strand breaks, elevation of circulating cytokine/chemokine levels, activation of iNOS and AKT-MAPK pathways, decreases in bone marrow cellularity and small intestinal villi and crypts, and burst of bacterial infection in every organ. As a result, cell death occurs; many organs lose function and then failed (Ledney and Elliott, 2010; Kiang et al., 2012; Fukumoto et al., 2013; Kiang and Ledney, 2013). Consequently, RI results in mortality (Kiang et al., 2010; Ledney and Elliott, 2010; Kiang et al., 2012; Kiang and Ledney, 2013). It is evident that radiation hits nuclus, cytosol, cells, tissues and organs. The detrimental responses are so complicated. This poly-traumatic complexity creates a hardship to identify plausible countermeasures for purpose of prevention or therapy. So, the nation is in need to develop effective drugs or means for taking care of RI, even though FDA has approved G-CSF (Neupogen®), pegylated G-CSF (Neulasta®) (Farese et al., 2013; Hankey et al., 2015), and Leukine for treating acute hemopoietic radiation syndrome (H-ARS).

RI remarkably increases circulating G-CSF (Kiang et al., 2010). It is generally thought that this rise is a possible self-defense mechanism, but its rise is too soon to play a role to repair the damage of radiation-sensitive organs including bone marrow (usually occurs within hours) and GI (usually occurs within days) after RI (Kiang et al., 2012; Kiang et al., 2014b). G-CSF and pegylated G-CSF (Peg-G-CSF) are utilized in hospitals for treating patients injured after radiotherapy or under the radionuclear accidents (Berger et al., 2006). It has been shown that G-CSF or Peg-G-CSF significantly attenuate not only the period of neutropenia and/or aplasia in victims suffered radiation but also strengthen recovery of neutrophil counts post anti-cancer therapy (Berger et al., 2006). They prime and/or stimulate neutrophils in order to enhance their function (Waselenko et al., 2004). The Peg-G-CSF is shown to have a longer and decent biological half-life than G-CSF (Molineux, 2004), therefore, this new formulation permits weekly administrations instead of daily injections; as known that daily injection causes distress and deters irradiated mice. Peg-G-CSF at the dose used in this report did not exhibit toxicity or harmful effects in mice.

Like the natural G-CSF, Peg-G-CSF enable 1) to stimulate the growth and division of myeloid progenitors, 2) to differentiate them into mature granulocytes, and 3) to induce mobilization of hematopoietic stem cell into the bloodstream from the bone marrow. Although our laboratory did not observe acceleration of wound healing (Kiang et al., 2010), Peg-G-CSF helps in wound healing (Badiavas et al., 2003) in addition to recovery of infection (Metcalf, 1990; Metcalf, 2007). When Peg-G-CSF was combined with erythropoietin and stem cell factors, this combinational therapy rescued a hospital technician who was exposed to a 4.5-Gy dose of radiation because this person entered a ^60^Co-irradiation therapy room by accident (Bertho et al., 2008). Other reports on victims who got exposed to radiation in radiological accidents and received G-CSF treatments have been documented (Singh et al., 2015).

In our B6D2F1/J mouse model (Kiang et al., 2014b; Kiang et al., 2014c), s.c. administrations of G-CSF alone (10 μg/kg, day 1 to day 14 once daily) after RI followed by p.o. administrations of levofloxacin (day 3 to day 21 once daily) and topical applications of gentamicin cream to the wound area (day 1 to day 10 once daily) significantly increased mouse survival by 25% after RI combined with wounding trauma. In a previous experiment, mice were injected with Peg-G-CSF, exhibiting an appreciably longer biological half-life in serum than G-CSF (Molineux, 2004) at 25 μg/mouse, s.c., once +24 h, +8 days, +15 days after 9.5 Gy (LD_50/30_) led to 100% 30-days survival post-RI, while vehicle-treated RI mice exhibited 30-days survival by 70%; significant recovery of monocytes, lymphocytes, and neutrophils in RI mice was observed (Kiang et al., 2014a). In the NHP model, treatment with Peg-G-CSF remarkably recovered neutrophil counts after irradiation at 6 Gy (Farese et al., 2012). Treatment with G-CSF effectively improved platelet and neutrophil counts after irradiation at 2 Gy in canine (MacVttie et al., 1990).

For public health emergency preparedness, there is an need for the most effective medical countermeasures to mitigate/treat RI victims. Because the survival increases in Peg-G-CSF-treated RI mice is around 30% above the vehicle-treated counterpart group (Kiang et al., 2014a), the object of this project was to investigate remedies that could enhance Peg-G-CSF efficacy in treating RI. Ghrelin was selected for this purpose because this co-therapy has been reported to reduce the RI-induced brain hemorrhage (Kiang et al., 2019; Gorbunov and Kiang, 2021) and Ghrelin alone was effective for other organ diseases (Wynne et al., 2005; Vasileious et al., 2013; Pereira et al., 2017; Fritz et al., 2020). Ghrelin is a peptide containing 28 amino acids; it is produced in the stomach during hunger and released into the blood stream to go to the hypothalamus for initiating the desire of food intake (Wynne et al., 2005; Vasileious et al., 2013; Pereira et al., 2017). It binds onto the growth hormone secretagogue receptor (GHS-R) coupling with G protein (Pereira et al., 2017). RI significantly increased interleukin (IL)-18 and enterocyte apoptosis in ileum after RI. The exogenous Ghrelin treatment reduced IL-18, decreased JNK activation and increased enterocytes and tight junction of ileum (Kiang et al., 2020). Moreover, the exogenous Ghrelin treatment significantly recovered lymphocytes, monocytes, and basophils, and enhanced increases in G-CSF in spleen samples after RI (Kiang et al., 2018). Therefore, it was of interest to investigate a hypothesis/concept, whether co-therapy of Peg-G-CSF and Ghrelin could enhance Peg-G-CSF’s efficacy to save lives and mitigate ARS after RI.

Herein, the thought that co-therapy with Ghrelin and Peg-G-CSF would demonstrate synergistic therapeutic effects for RI was hypothesized. Therefore, our report proves the hypothesis/concept that this co-therapy indeed enhanced survival probably due to the combined effects of body weight recovery, platelets recovery, inhibition of splenomegaly and injury of bone marrow and intestine.

## Methods

### Experimental Design

B6D2F1/J female mice were divided randomly into eight groups (*N* = 20–42 per group, conducted in four separated experiments): 1) sham + vehicles, 2) radiation + vehicles, 3) sham + Peg-G-CSF + Ghrelin, 4) radiation + peg- G-CSF + ghrelin, 5) sham + Peg-G-CSF, 6) Radiation + Peg-G-CSF, 7) sham + Ghrelin, and 8) radiation + Ghrelin.

### Animals

B6D2F1/J mice were purchased from the Jackson Laboratory, Bar Harbor, ME. Only female mice were used in this study based on the previous studies conducted in this laboratory (Kiang et al., 2010; Ledney and Elliott, 2010). These mice were 14–16 weeks old with an average weight of 24–25 g at the time of irradiation. Male mice were not used here because they are more aggressive to each other when they are housed together resulting in unnecessary injuries as described previously (Ledney and Elliott, 2010). Upon arrival, all mice were acclimated for 7 days. They were housed in plastic microisolator cages on hardwood chip bedding. They were maintained in the vivarium located at the Armed Forces Radiobiology Research Institute accredited by the Association for Assessment and Accreditation of Laboratory Animal Care International (AAALAC International). Acidified tap water as well as commercial rodent chow were made available *ad libitum*. A 12 h 0,600 (light) to 1800 (dark) full-spectrum lighting cycle was used in animal holding rooms, where the temperature was maintained at 21°C. With at least 10 changes/h of 100% conditioned fresh air, the relative humidity was 10%. Commercial rodent chow was Harlan Teklad Rodent Diet 8,604. Acidified water was with pH = 2.5–3.0 in order to inhibition of opportunistic infections.

The Armed Forces Radiobiology Research Institute (AFRRI) Institutional Animal Care and Use Committee (IACUC) approved all animal procedures. The recommendations and guidance of the American Veterinary Medical Association were followed when mice received euthanasia.

### Gamma Irradiation

Mice were given 9.5 Gy (Kiang et al., 2014b; Kiang et al., 2019; Kiang et al., 2020) ^60^Co γ-photon radiation (whole-body bilateral; 0.4 Gy/min; Kiang et al., 2019; Kiang et al., 2020). The exposure time for each irradiation was determined from the mapping data, with application of corrections due to the ^60^Co decay plus the little variation in the mass energy absorption coefficients for water and soft tissues. The field was uniform within ±2%. With an ionization chamber adjacent to the mouse towers and calibration of dose to the midline soft tissue of mice, the accuracy of the actual dose delivery for each run was verified and recorded.

### Preparation and Administration of Pegylated G-CSF and Ghrelin

Pegylated G-CSF [(Peg-G-CSF), aka Neulasta® and pegfilgrastim; NDC: 555-13-019001] is a polyethylene glycol pharmaceutical-formulated-grade drug. It was obtained from the AmerisourceBergen Corporation (Valley Forge, PA). Peg-G-CSF-treated mice were s.c. injected at 1,000 μg/kg in a volume of 0.2 ml 1 day, 8 days, and 15 days after RI (Kiang et al., 2014b; Kiang et al., 2019). This dose was based on the clinical human dose utilized for the purpose of subcutaneous auto-injection by patients. Neulasta® was commercially supplied in 0.6 ml prefilled syringes. Six mg Peg-G-CSF in a sterile, clear, colorless, preservative-free solution containing 30 mg sorbitol, 0.02 mg sodium, 0.02 mg polysorbate 20, and 0.35 mg acetate in water for injection, USP was in each syringe. The vehicle-treated mice received 0.2 ml of vehicle containing 30 mg sorbitol, 0.02 mg sodium, 0.02 mg polysorbate 20, and 0.35 mg acetate in 0.6 ml water (Kiang et al., 2014b; Kiang et al., 2019).

Ghrelin, obtained from Phoenix Pharmaceutical (Burlingame, CA), was subcutaneously (s.c.) administered at three doses of 113 μg/kg in a volume of 0.2 ml 24 h, 48 h, and 72 h after sham or RI. The dose was calculated based on a publication (Shah et al., 2009) and utilized previously (Kiang et al., 2018; Kiang et al., 2020). The vehicle given to control mice was sterile 0.9% sodium chloride solution for injection, USP (Kiang et al., 2020).

### Thirty-Day Survival

After irradiation, mice (*N* = 20–42 per group) were closely monitored for 30 days by the research staff in addition to the regular health checks by vivarium staff. During the 30 days monitoring period, the AFRRI IACUC Policy 020 was followed (Koch et al., 2016).

### Body Weight Measurement

Mouse body weights were measured right after irradiation (considered to be day 0). Then their body weights were measured on days 1, 3, 7, 14, 21, and 28.

### Measurement of Daily Water Consumption

Mice were housed in four per cage. The drinking bottle was placed on the top of each cage. Mice received drinking water that was contained in a steam-sterilized graduated bottle. A sipping tube with a metal ball inside was connected to the drinking bottle to prevent water leakage. For the first 7 days after irradiation, the volume of water drunk daily was measured. Then, we calculated the average volume of water drunk by each mouse on each day in each cage (Kiang et al., 2014a). Water consumption by each mouse per day was presented as mL/animal/day in the Figure.

### Blood Collection, Peripheral Blood Cell Count, Serum Preparation, and Tissue Collection

On day 30 after RI, mice were under deep isoflurane anesthesia via cardiac puncture into a 1 ml-syringe to collect blood samples from each mouse in each group. Then, 300 µL blood was placed into the EDTA-containing microtube and maintained in a rotator. Blood cell counts were assessed with the ADVIA 2120 Hematology System (Siemens, Deerfield, IL). Differential analysis was carried out with the peroxidase method and the light scattering techniques following the manufacturer’s manual

The rest of blood in the 1-ml syringe was put into a microtube with serum separator additive for serum preparation. After at least 30 min coagulation at room temperature, sera were collected after centrifugation at 10,000× g for 10 min, and immediately stored at −80°C for future analysis.

Cervical dislocation was performed after blood draw. Sternums, femurs, ileums, and spleens were collected. The number of animals used for blood samples and tissue samples was up to six per group.

### Bone Marrow Cell Count

On day 30 after irradiation, two femurs from each mouse were collected. Bone marrows were flushed out using 3 ml 1x phosphate-buffered saline (PBS) buffer twice. The marrows were then centrifuged at 800× g for 10 min (Sorvall Legend XTR Centrifuge, Thermo Scientific) and the pellets were re-suspended in 10 ml 1x ACK buffer (Invitrogen, Grand Island, NY) and centrifuged at 800× g for 10 min. The resulted pellets were re-suspended in 10 ml 1x PBS buffer. The cell suspensions were then placed in Countess™ cell-counting-chamber slides (Invitrogen) and counted using a Countess™ automated cell counter (Invitrogen) (Kiang et al., 2014b; Kiang et al., 2018). Bone marrow cells were finally centrifuged again at 800x g for 10 min. The cell pellets were stored in −80°C until the future analysis. Bone marrow cells were presented as cells/femur.

### Spleen Weight and Splenocyte Count

On day 30 after irradiation, spleens were collected from each surviving mouse (*N* = 6 mice per group). Spleens were weighed first. Then, each spleen was inserted into a plastic bag containing 10 ml 1× Hank’s Balanced Salt Solution (HBSS; Invitrogen, Grand Island, NY), homogenized using Seward Stomacher® 80 (Thermo Scientific), and poured through a 70 mm cell strainer (BD Falcon, Bedford, MA). The bag and strainer were rinsed with 15 ml 1x HBSS again. The fluid with Splenocytes in the tube was then centrifuged at 800× g (Sorvall Legend XTR Centrifuge, Thermo Scientific) for 10 min. The pellets were resuspended in 10 ml 1× ACK lysis buffer (Invitrogen) for 10 min at 37°C to lyse RBCs, mixed by vertexing every 5 min, and then centrifuged at 800× g for 10 min. Splenocyte pellets were collected, resuspended in 10 ml 1× phosphate-buffered saline (PBS). The cell suspension was placed in Countess™ cell-counting-chamber slides (Invitrogen, Eugene, Oregon) and counted with the Countess automated cell counter (Invitrogen). Splenocyte suspension was finally centrifuged again at 800x g for 10 min. The cell pellets were stored in −80°C until the future analysis. The spleen weight was presented in mg and splenocyte counts were presented as cells/spleen.

### Histological Examination of Bone Marrow and Intestine

On day 30 after irradiation, sternum and ileum specimens were collected from each mouse in each group (*N* = 4 mice per group). Sternums and ileum were rinsed in cold saline and then immediately placed in 10% phosphate-buffered formalin. They later were embedded in paraffin. Sternums were sectioned longitudinally and ileums were sectioned transversely. They were stained with Hematoxylin and Eosin. Using Zeiss Axioscan.Z1, the histology slides were scanned and stored. Using Zen two software (Zeiss Company, Thornwood, NY), fat cells and megakaryocytes in each sternum slide at a ×40 magnification were counted in four fields. Villus height, villus width, crypt depth, and crypt numbers in each ileum slide at ×20 magnification were counted. The mucosal injury score represented each slide was given (Kiang et al., 2020) To briefly summarize the standard of scores (Kiang et al., 2020): grade 0 = normal mucosa; grade 1 = development of subepithelial spaces near the tips of the villi with capillary congestion; grade 2 = extension of the subepithelial space with moderate epithelial lifting from the lamina propria; grade 3 = significant epithelial lifting along the length of the villi with a few denuded villus tips; grade 4 = denuded villi with exposed lamina propria, dilated capillaries and reduced crypt depth and counts; and grade 5 = disintegration of the lamina propria, hemorrhage, and ulceration.

### Statistical Analysis

We present our data as the mean ± S.E.M. Using a Kaplan-Meier curve and the log rank test for each survival experiment in which 20-42 mice per group were individually tested. One-way ANOVA, two-way ANOVA, studentized-range test, and Student’s t-test were used in comparison of groups for hematological analysis (*N* = 6 per group) and histopathological analysis (*N* = 4 per group). The statistical significance was considered when *p* value was less than 5%.

## Results

### Ghrelin Enhances the Peg-G-CSF Therapy-Induced Survival Improvement After Lethal Irradiation

**FIGURE 1:**
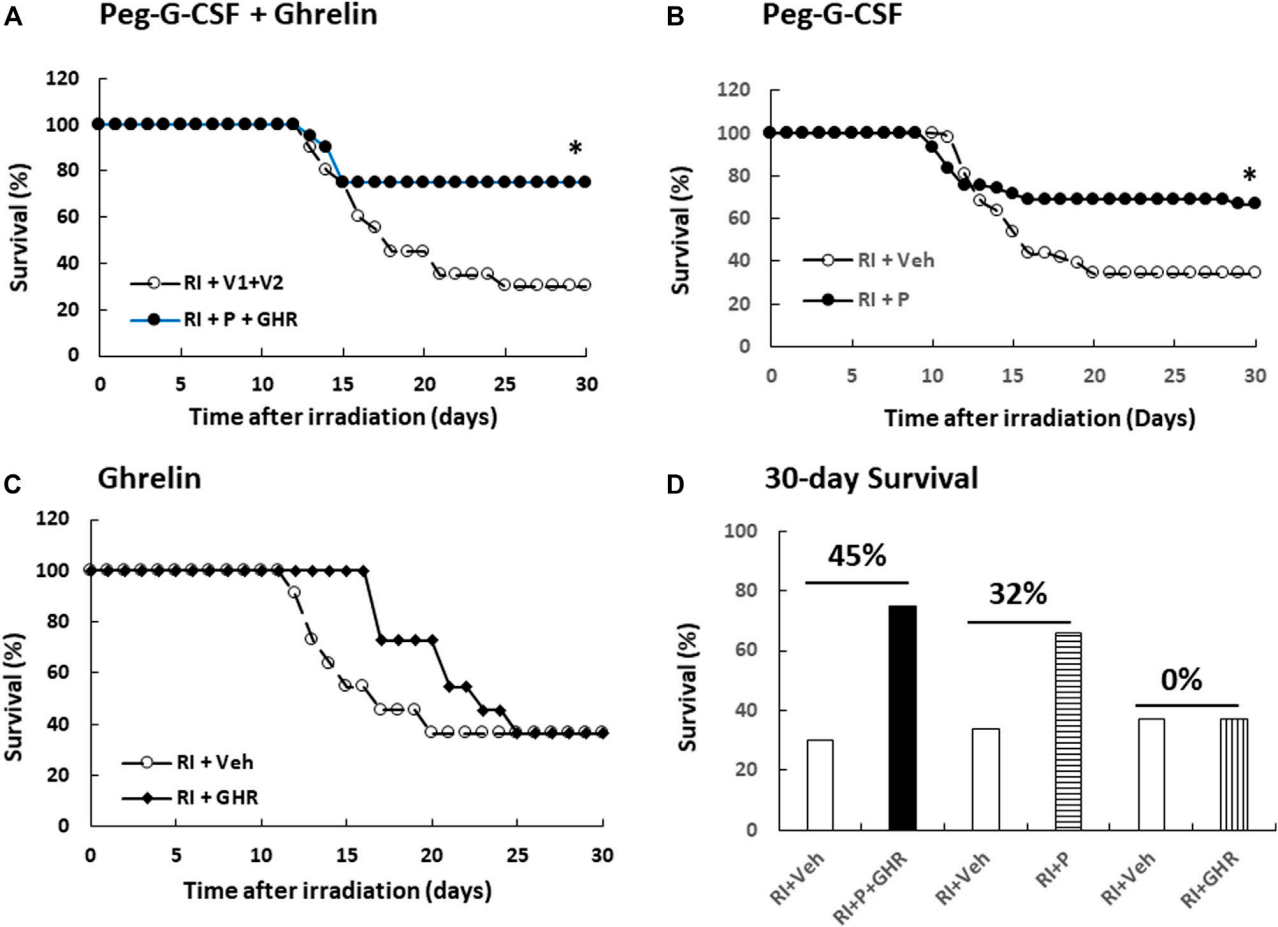
Co-therapy of Peg-G-CSF and Ghrelin reduces mortality after irradiation (RI). *N* = 20–42 per group. Non-irradiated mice treated vehicle, individual drug or combination of the two were survived by 100%. On the **(A)** panel, *p < 0.05 vs. RI + V1+V2. On the **(B)** panel, *p < 0.05 vs. RI + Veh, **(C)** Survival curves with Ghrelin treatment. **(D)** Numbers above horizontal lines on the top of two bars represent the percentage of survival differences between the drug-treated group and its respective vehicle group. RI: 9.5 Gy; V1, vehicle for Peg-G-CSF; V2, vehicle for Ghrelin; Veh, vehicle; P, Peg-G-SF; GHR, Ghrelin.

Radiation is known to decline survival (Kiang et al., 2010). As shown in Figure 1A, RI reduced survival down to 30%, therapy of Peg-G-CSF and Ghrelin further increased 30-day mouse survival to 75% (*p* < 0.05 vs. RI + V1+V2). In RI mice treated with either Peg-G-CSF (Figure 1B) or Ghrelin (Figure 1C) alone, % survival increases above vehicle group was 32% and 0% (*p* < 0.05 vs. RI + *p* vs. RI + GHR), respectively (Figure 1D). The non-irradiated mice treated with either Peg-G-CSF, Ghrelin, or combination of the two survived by 100%, suggesting the doses used were safe, although Ghrelin has been reported to promote fear, anxiety- and depression-like behaviors in rodents (Fritz et al., 2020).

### Peg-G-CSF and Ghrelin Therapy Mitigates Body Weight Loss After Lethal Irradiation

**FIGURE 2:**
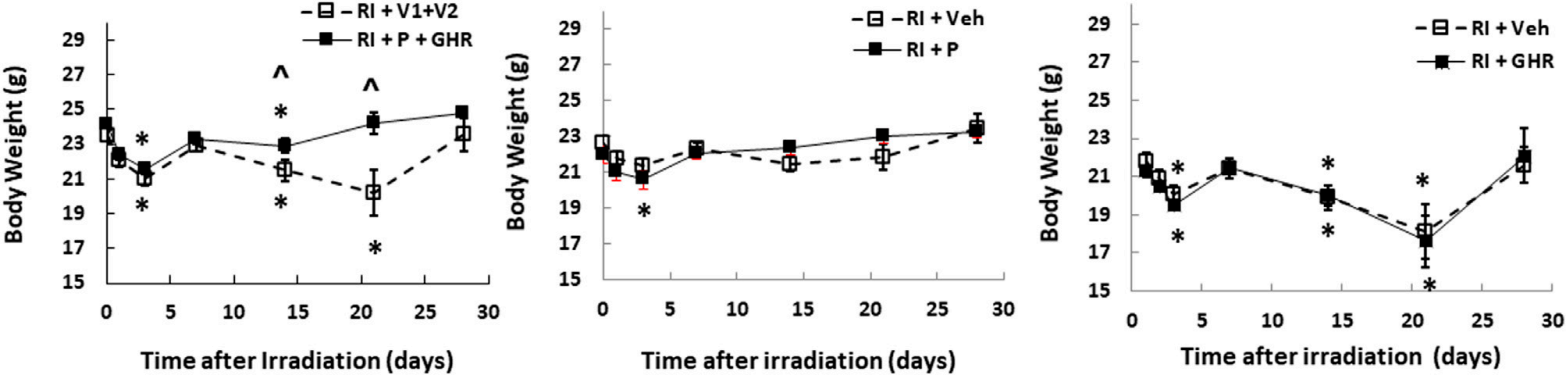
Co-therapy of Peg-G-CSF and Ghrelin mitigates the RI-induced body-weight loss. *N* = 20–22 per group. Data are shown as mean ± sem. *p < 0.05 vs. day 0; ^*p* < 0.05 vs. RI + *p* + GHR. RI: 9.5 Gy; V1, vehicle for Peg-G-CSF; V2, vehicle for Ghrelin; P, Peg-G-SF; GHR, Ghrelin.

Radiation lowers the body weight beginning on the 3rd day after lethal radiation exposure (Kiang et al., 2010). Therefore, we measured the body weight after RI. RI significantly decreased the body weight on day 2, bounced back on day 7, then decreased again on day 14, continued to decrease to reach the nadir on day 21, but began to gain the weight back (Figure 2). Co-therapy of Peg-G-CSF and GHR attenuated the body-weight loss on days 14 and 21 in the RI mice. Treatment with either Peg-G-CSF or GHR alone had no improvement on the RI-induced body weight loss.

### Peg-G-CSF and Ghrelin Therapy Reduces Water Consumption After Radiation Injury

**FIGURE 3:**
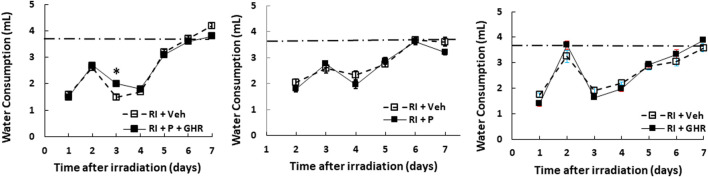
Co-therapy of Peg-G-CSF and GHR increases water consumption after irradiation (RI). *N* = 20–22 per group. The non-irradiated mouse daily water consumption was 3.7 ± 0.1 ml (*N* = 20). Data are shown as mean ± sem. *p < 0.05 vs. RI + Veh. RI: 9.5 Gy; Veh, vehicle for Peg-G-CSF and vehicle for Ghrelin; P, Peg-G-SF; GHR, Ghrelin.

The mouse daily water consumption is 3–4 ml. It is evident that RI significantly reduced water consumption. Figure 3 shows that in comparison to the sham group (3.7 ± 0.1 ml), RI mice significantly reduced water consumption by 60% on day 1 (1.6 ± 0.1 ml, *p* < 0.05 vs. sham), but went back to baselines on the 7th day. Co-therapy of Peg-G-CSF and Ghrelin increased it in RI mice on day 3, whereas treatment with either Peg-G-CSF or GHR alone had no improvement on the RI-induced water consumption inhibition.

### Peg-G-CSF and Ghrelin Therapy Mitigates Bone Marrow Histopathology

**FIGURE 4:**
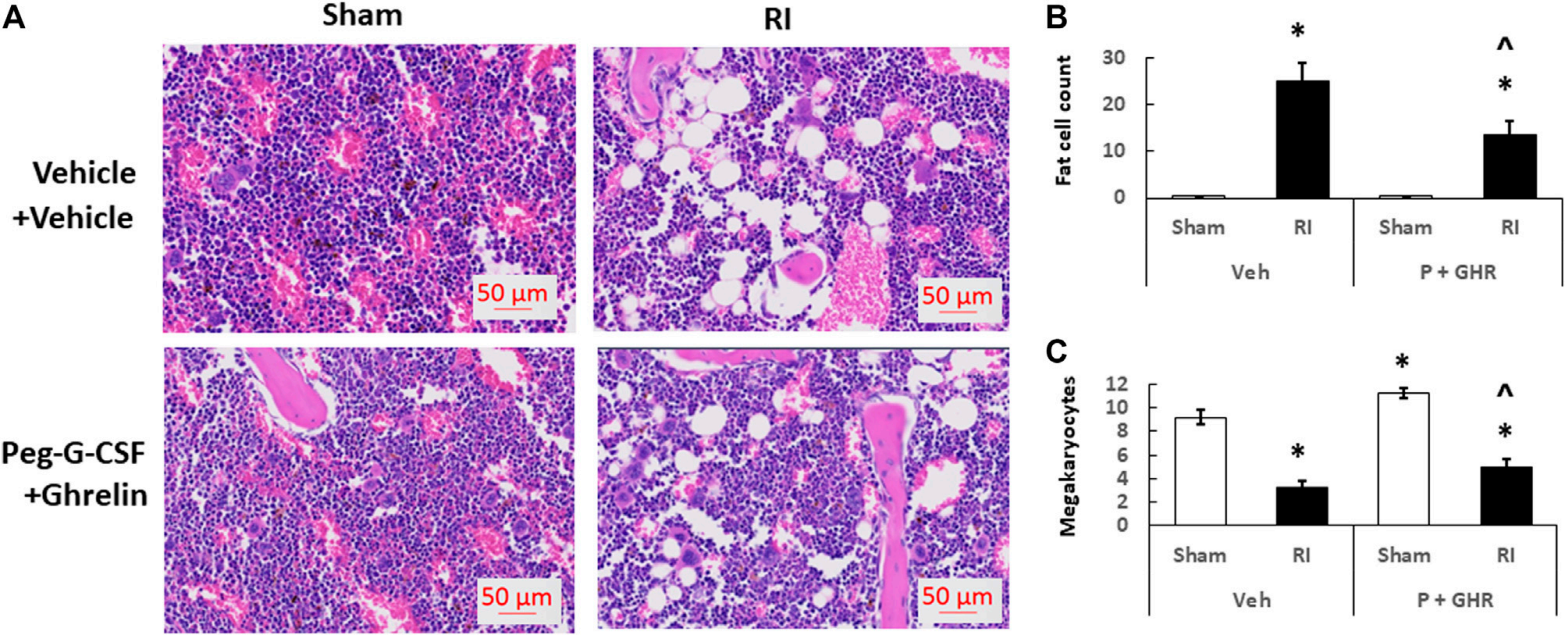
Co-therapy of Peg-G-CSF and GHR recovers bone marrow cellularity after irradiation (RI). Data are shown as mean ± sem. **(B,C)**. Bone marrow histology slides on day 30 post-RI stained with hematoxylin and eosin **(A)**. N = 4 per group. Fat cells **(B)** and megakaryocyte counts **(C)** at four fields with ×20 magnification were measured. *p < 0.05 vs. Sham + Veh; ^*p* < 0.05 vs. RI + Veh. RI: 9.5 Gy; Veh,vehicle for Peg-G-CSF and vehicle for Ghrelin; P, Peg-G-SF; GHR, Ghrelin.

Figure 4 depicts that on day 30 after RI, RI significantly reduced the bone marrow cellularity in vehicle-treated mice, supported by increasing counts of adipocyte (Figure 4B) and decreasing megakaryocytes counts in bone marrow histology slides (Figure 4C). Co-therapy of Peg-G-CSF and Ghrelin immensely mitigated the number of adipocytes (Figure 4B) and partially recovered the number of megakaryocytes (Figure 4C) in RI mice. It is interestingly noted that the co-therapy significantly elevated megakaryocyte counts in sham mice as well (Figure 4C).

### Peg-G-CSF and Ghrelin Therapy Mitigates Bone Marrow Cell Counts After Radiation Injury

**FIGURE 5:**
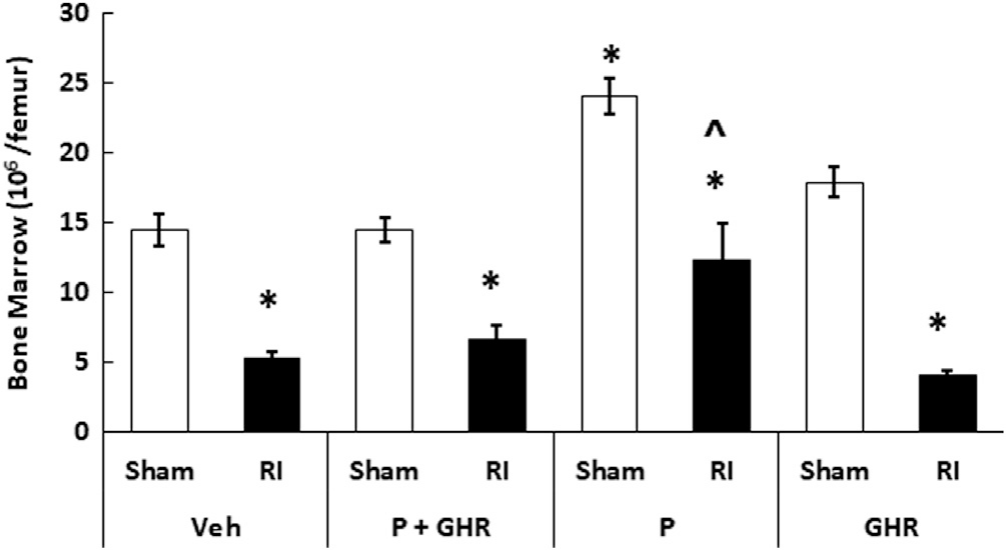
Co-therapy of Peg-G-CSF and Ghrelin increases bone marrow cell counts after irradiation (RI). Bone marrow cells were collected from two femurs of each mouse. *N* = 6 per group. Data are shown as mean ± sem. *p < 0.05 vs. Sham + Veh; ^*p* < 0.05 vs. RI + Veh, RI + P + GHR, and RI + GHR. RI: 9.5 Gy; Veh, vehicle for Peg-G-CSF and vehicle for Ghrelin; P, Peg-G-SF; GHR, Ghrelin.

Figure 5 depicts that on day 30 after RI, the bone marrow cellularity was significantly reduced in mice treated with vehicles. Co-therapy of Peg-G-CSF and Ghrelin induced a significant increase in the cell counts in RI mice. The treatment with Peg-G-CSF alone significantly elevated the cell counts in sham mice and RI mice, while the treatment with Ghrelin alone did not.

### Peg-G-CSF and Ghrelin Therapy Mitigates White Blood Cell Loss but Not Red Blood Cell Loss After Radiation Injury in Peripheral Blood

**FIGURE 6:**
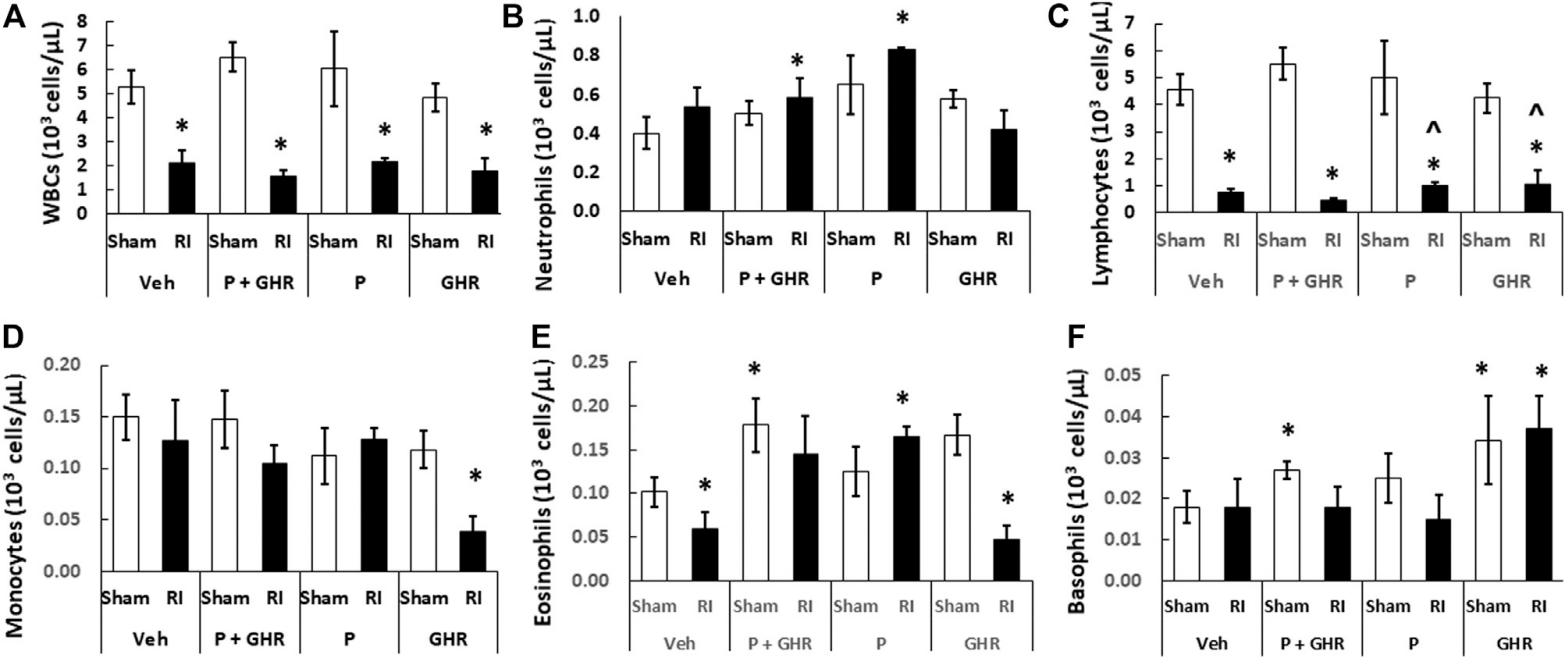
Co-therapy of Peg-G-CSF and Ghrelin significantly increases neutrophils, eosinophils and basophils after irradiation (RI). **(A–F)** WBCs of blood samples collected on day 30 post-RI. *N* = 6 per group. Data are shown as mean ± sem. *p < 0.05 vs. Sham + Veh; ^*p* < 0.05 vs. RI + Veh. RI: 9.5 Gy; Veh, vehicle for Peg-G-CSF and vehicle for Ghrelin; P, Peg-G-CSF; GHR, Ghrelin.

Radiation is known to deplete WBCs (Kiang et al., 2010). As shown in Figure 6, in RI mice, combined treatment with Peg-G-CSF and Ghrelin significantly mitigated neutrophils and eosinophils. Treatment with Peg-G-CSF alone increased neutrophils, lymphocytes and eosinophils; treatment with Ghrelin alone significantly elevated lymphocytes and basophils in RI mice.

**FIGURE 7:**
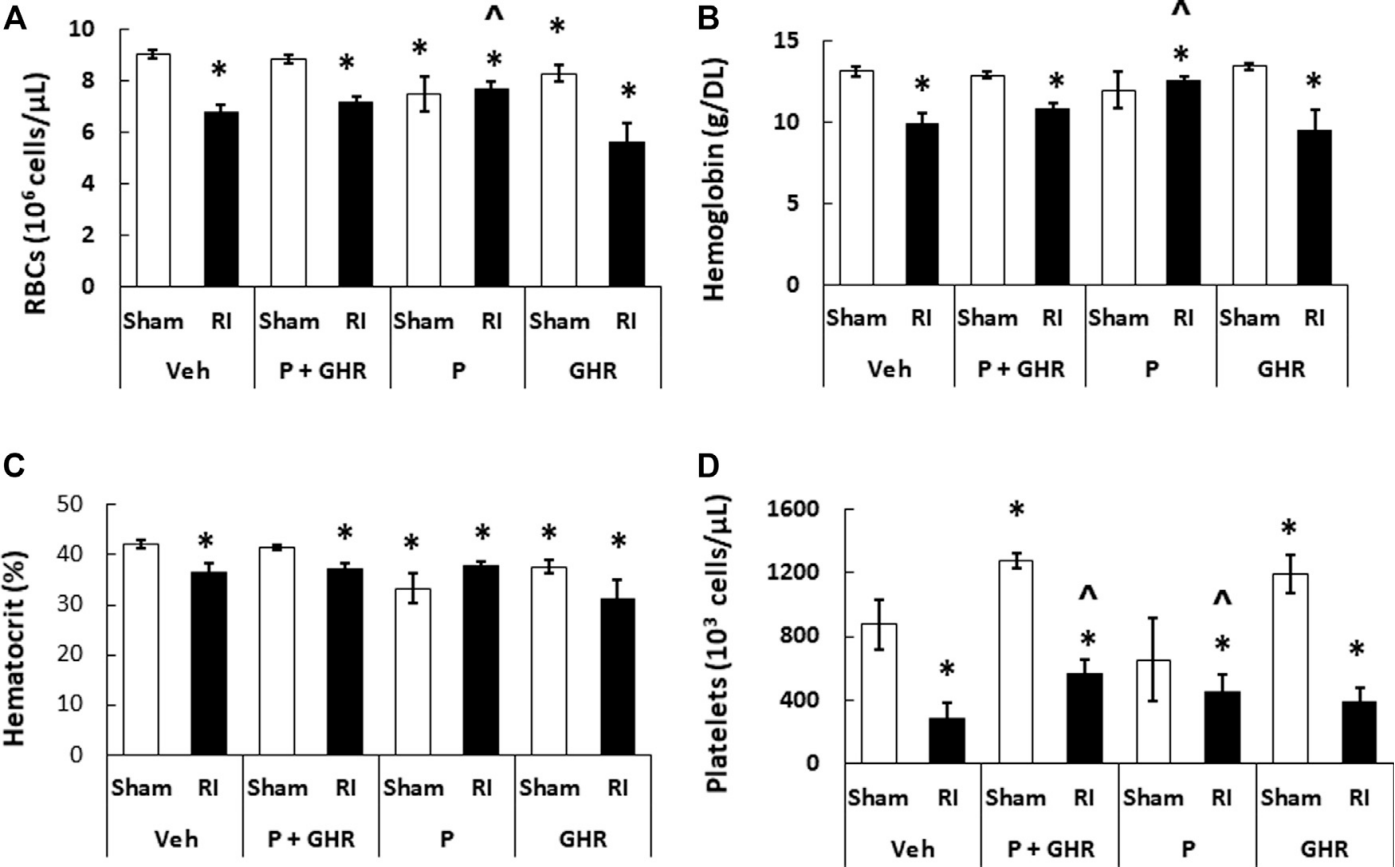
Co-therapy of Peg-G-CSF and Ghrelin significantly mitigate platelet counts but not RBCs, hemoglobin and hematocrit after irradiation (RI). RBCs **(A)**, Hemoglobin **(B)**, Hematocrit, **(C)** and Platelets counts **(D)** in blood samples of sham and RI mice on the 30th day were measured. *N* = 6 per group. Data are shown as mean ± sem. *p < 0.05 vs. Sham + Veh; ^*p* < 0.05 vs. RI + Veh. RI: 9.5 Gy; Veh, vehicle for Peg-G-CSF and vehicle for Ghrelin; P, Peg-G-CSF; GHR, Ghrelin.

RI is known to deplete RBCs (Kiang et al., 2010). As shown in Figure 7, treatment with Peg-G-CSF and Ghrelin did not mitigate reduction of RBC numbers, hemoglobin levels and hematocrit readings. It is noted that Peg-G-CSF treatment alone significantly mitigated reduction of RI-induced RBC and hemoglobin, whereas treatment with Ghrelin did not improve the RBC counts, hemoglobin levels and hematocrit readings after RI.

### Peg-G-CSF and Ghrelin Therapy Mitigates the Radiation Injury-Induced Platelet Loss

Figure 4 shows that co-therapy of Peg-G-CSF and Ghrelin induced a significant mitigation of the megakaryocyte loss in surviving RI animals. The number of platelets in peripheral circulation was counted because megakaryocytes are the precursors of circulatory platelets. Peg-G-CSF and Ghrelin combined therapy significantly increased platelets in sham mice and RI mice, which is fully correlated with the increased megakaryocytes in bone marrow samples of sham and RI mice (Figure 7D). Treatment with Peg-G-CSF alone mitigated the RI-induced platelet loss while treatment with Ghrelin alone increased platelets in sham mice but not in RI mice.

### Peg-G-CSF and Ghrelin Therapy Inhibits the Radiation Injury-Induced Splenomegaly

**FIGURE 8:**
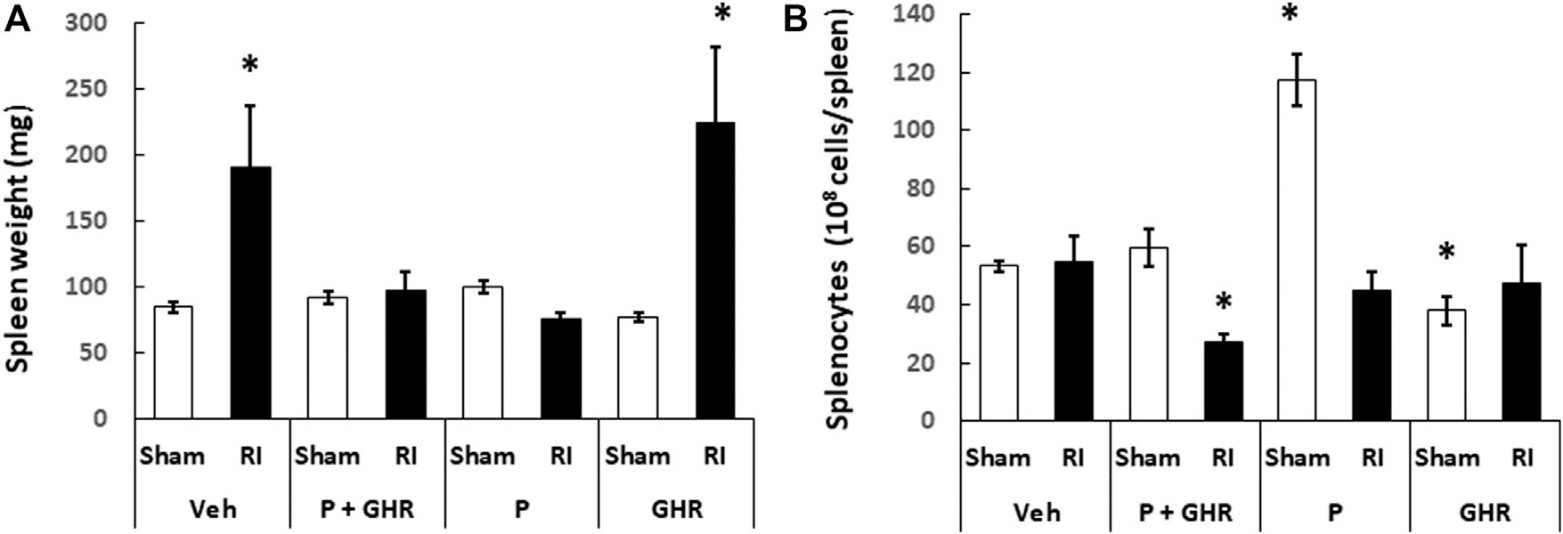
Co-therapy of Peg-G-CSF and Ghrelin inhibits the radiation-induced splenomegaly. Spleen weight **(A)** and splenocytes **(B)** in sham and RI mice on the 30th day were measured. *N* = 6 per group. Data are shown as mean ± sem. *p < 0.05 vs. Sham + Veh; RI: 9.5 Gy; Veh, vehicle for Peg-G-CSF and vehicle for Ghrelin; P, Peg-G-SF; GHR, Ghrelin.

Spleen is important for survival after lethal RI (Jacobson, 1952). Figure 8A shows that RI resulted in splenomegaly. Treatment with Peg-G-CSF and Ghrelin fully inhibited this splenomegaly from occurring. Treatment with Peg-G-CSF alone also inhibited it. In contrast, treatment with Ghrelin did not, which strongly suggests that Peg-G-CSF is the primary drug to lead to this inhibition.

Although RI induced splenomegaly, Figure 8B depicts that splenocyte counts on day 30 after RI had returned to basal levels. Treatment with Peg-g-CSF and Ghrelin reduced the splenocyte counts in RI mice. Treatment with Peg-G-CSF alone dramatically increased the counts in sham mice and recovered the counts in RI mice. On the other hand, treatment with Ghrelin alone lowered splenocytes in sham mice but not in RI mice.

### Peg-G-CSF and Ghrelin Therapy Mitigates Intestine Histopathology

**FIGURE 9:**
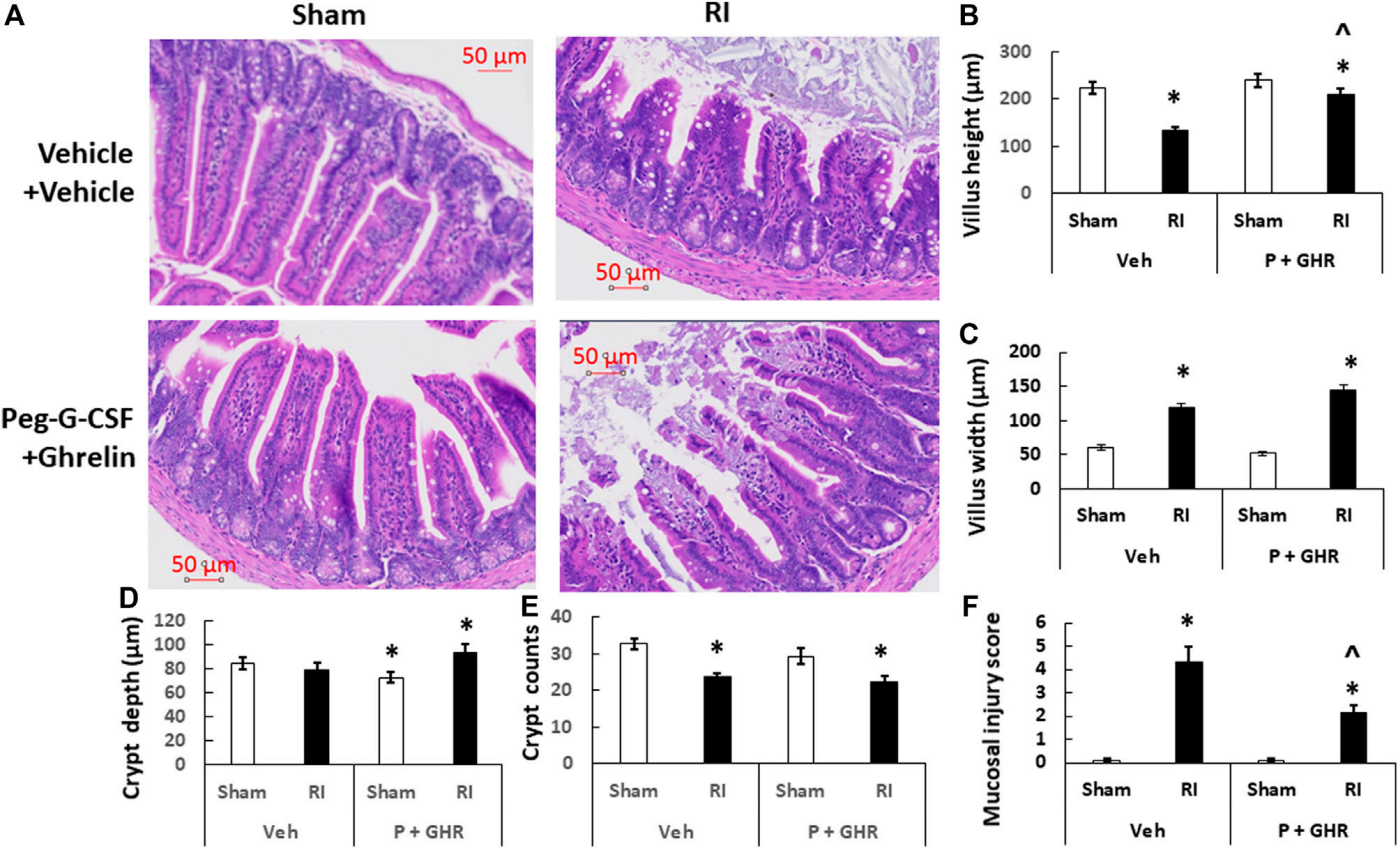
Co-therapy of Peg-G-CSF and Ghrelin mitigates the radiation-induced ileal injury. **(A)** Histology slides stained with H&E of ileums collected on the 30th day post-RI. *N* = 4 per group. **(B–F)** Parameters of ileums including mucosal injury scores, crypt counts and depth, and villus heights and width were assessed. Data are shown as mean ± sem. *p < 0.05 vs. Sham + Veh; ^*p* < 0.05 vs. RI + Vehicle. RI: 9.5 Gy; Veh, vehicle for Peg-G-CSF and vehicle for Ghrelin; P, Peg-G-CSF; GHR, Ghrelin.

RI is known to injure intestine (Kiang et al., 2010). Figure 9 shows that on day 30 after RI, decreases in villus height, increases in villus width, decreases in crypt counts and increases in mucosal injury score were observed. Treatment with Peg-G-CSF and Ghrelin increased villus height and crypt depth and decreased the mucosal injury score, although the RI-induced increases in villus width and decreases in crypt counts had remained the same.

## Discussion

The present paper reports that RI noticeably increased lethality accompanied by body-weight loss and reduced water consumption in mice. These results are in agreement with previous reports in rat (Alpen and Sheline, 1954; Valeriote and Baker, 1964), guinea pig (Korlof, 1956), dog (Brooks, 1952), swine (Baxter et al., 1953), and mice (Ledney et al., 1981; Ledney et al., 1992; Jacob et al., 2010; Ledney and Elliott, 2010; Palmer et al., 2011; Kiang et al., 2012; Kiang and Ledney, 2013; Islam et al., 2015; Kiang and Olabisi, 2019). RI is known to cause massive cellular damage, macro/microcirculation failure, fluid imbalance, immune system inhibition, and acute myelosuppression, thereby, as a result, leading to disruption of vital organ functions. Then, multiple organ dysfunction (MOD) and multiple organ failure (MOF) are manifested and death occurs after irradiation (Brooks et al., 1952; Baxter et al., 1953; Korlof, 1956; Kiang and Olabisi, 2019).

Drugs such as 5-androstenediol (Grace et al., 2012; Whitnall et al., 2002), G-CSF (Farese et al., 2013; Kiang et al., 2014b), Peg-G-CSF (Kiang et al., 2014b; Hankey et al., 2015), and captopril (Islam et al., 2015), were reported to be effective in reducing the lethal RI-induced mortality. Among them, Neupogen (G-CSF) and Neulasta (Peg-G-CSF) are FDA-approved. Even though, the efficacy of either G-CSF or Peg-G-CSF is approximately 30% survival improvement after lethal irradiation exposure. Thus, such a combined therapy of Peg-G-CSF and Ghrelin was attempted and found to result in 75% survival and significantly mitigated body-weight loses after RI, suggesting that keeping up the body weight plays an essential role in this co-therapy.

Peg-G-CSF exhibits an appreciably longer biological half-life in serum than G-CSF (Molineux, 2004). So, daily injections can be avoided as well as eliminating the injection-associated stress. Nonetheless, the Peg-G-CSF’s survival efficacy remained similar to G-CSF after irradiation (Kiang et al., 2014b). The Peg-G-CSF survival efficacy data after RI is consistent with that observed in nonhuman primates (Hankey et al., 2015).

RI significantly reduced the bone marrow cell counts as shown by significant decreases in megakaryocyte counts plus significant rises in fat cells (Figure 4). The observations are similar to our previous reports (Kiang et al., 2017; Wang et al., 2021). The increase in platelets (Figure 7D) is further confirmed with the increases in megakaryocytes in bone marrow. Because the RI-induced hemorrhage occurs, increases in platelets by the co-therapy become critical for healing and subsequent survival (Kiang et al., 2019). Moreover, this increase is primarily contributed by the treatment with Ghrelin but not Peg-G-CSF (Figure 7D).

RI significantly reduced WBC counts even on day 30 (Kiang et al., 2010; Kiang et al., 2012). mainly due to very low counts of eosinophils and lymphocytes (Figure 6). The data are similar to the finding obtained in nonhuman primates that were irradiated prior to treatment with peg-megakaryocyte growth and development factor in combination with G-CSF therapy (Farese et al., 1996). Peg-G-CSF is known to stimulate myeloid progenitors to proliferate, to differentiate, to become mature granulocytes, to get mobilized into the bloodstream from the bone marrow, and most importantly to make mature neutrophils effective in combating RI-induced infection (Metcalf, 1990; Metcalf, 2007) and wound healing (Badiavas et al., 2003). We think, that the co-therapy increased platelets in sham animals due to Ghrelin, but in RI mice due to both Peg-G-CSF and Ghrelin (Figure 6), further reinforced by the recovery of bone marrow cellularity (Figure 5).

Reports from our laboratory and other laboratories indicate that Peg-G-CSF treatment alone in mice (Kiang et al., 2014b), G-CSF administration in canines (MacVittie et al., 1990), and an IL-3/G-CSF receptor agonist in nonhuman primates (MacVittie et al., 2000) increased platelet counts after RI. In this study, we found the co-therapy effectively increased platelet counts after RI. These data suggest that recovery of platelets may contribute at least partially to the RI mouse 30-days survival. G-CSF administration to healthy humans is known to trigger endothelial cell activation to lead to an inflammatory process so as to increase platelet counts (Ihara et al., 2008). However, we postulate that Peg-G-CSF administration to healthy mice is incapable of triggering such process resulting in no visible induction of thrombopoiesis in sham mice. On the other hand, Ghrelin enables to increase thrombopoiesis in sham mice.

On day 30 after RI, significantly low hematocrit readings, hemoglobin levels, and RBC counts were remained in surviving mice (Figure 7). Despite the RI-induced decreases were fully recovered in Peg-G-CSF treated mice, in Ghrelin-treated mice and co-treated mice no such a recovery was found, suggesting increases in erythropoietin production by Peg-G-CSF may have been antagonized by Ghrelin. It warrants further exploration in this line.

Jacobson (1952) reported that mice with protected spleen survived from the lethal irradiation, suggesting spleen plays a key role for survival. Herein, we observed RI induced a spleen enlargement without altering the splenocyte counts. The co-therapy inhibited this enlargement, which was attributed primarily by Peg-G-CSF‘s inhibition. Ghrelin treatment alone did not block the RI-induced splenomegaly (Figure 8). We also found that the co-therapy reduced splenocytes after RI. In contrast, treatment with either one did not. It is elusive and worth to explore further.

Table 1 summarizes observations of survival, body weight, WBCs, RBCs, platelets, spleen weigh and splenocytes. In the presence of Peg-G-CSF and Ghrelin, mitigation of body weight loss, neutrophil depletion, and platelet reduction are important for survival after RI. Additionally, literature documents that RI at the LD_50/30_ dose damages gastrointestinal system (Kiang and Olabisi, 2019).

**TABLE 1:** Treatment with Peg-G-CSF alone, Ghrelin alone, or the combination of both impacts survival, body weight, WBCs, RBCs, platelets, spleen weight and splenocytes after RI. Survival, body weight, numbers of WBCs, RBCs, and platelets, spleen weight, and splenocytes were reduced after RI and remained low 30 days later. Comparisons are made from data pooled from three separate experiments.

	Increases in survival above RI + Veh (%)	Body weight	WBCs	RBCs	Platelets	Spleen weight	Splenocytes
Peg-G-CSF	32	↓	↓	↑	↓	↓	↑
Ghrelin	0	↓	↓	↓	↑	↑	↑
Peg-G-CSF + ghrelin	45	↑	↓	↓	↑	↓	↓

RI, radiation injury; Veh, vehicles; ↑, increase; ↓, decrease.

Our laboratory has been investigated ileum because the section near caecum is empty and easy for histology examination and analysis. RI reduced the villus height and crypt counts, and increased villus swelling and mucosal injury (Figure 9). This co-therapy was effective to combat these detrimental outcomes, very similar to the Ghrelin treatment alone (Kiang et al., 2020) whereas treatment with Peg-G-CSF alone failing to improve the ileum injury has been demonstrated (Wang et al., 2021). These results, taking together with previous observations with either bone marrow hemopoietic stem cells resulting in more than 90% survival (Ledney and Elliott, 2010) or mesenchymal cells failing in survival improvement (Kiang and Gorbunov, 2014), suggest that Ghrelin treatment primarily contributes to the ileum recovery including improvement of tight junctions, enterocytes, sepsis and cell survival by inhibiting IL-18 and bcl-2-like protein 4 (BAX) signals (Kiang et al., 2020), whereas Peg-G-CSF treatment primarily repaires bone marrow by increasing granulocyte, erythrocyte, monocyte, megakaryocyte (GEMM) colonies (Wang et al., 2021). We consider Peg-G-CSF and Ghrelin target bone marrow and intestine, respectively, leading to this enhancement in survival.

To elucidate the underlying molecular mechanisms, tissues from both surviving and moribund animals at early time points are desirable for investigation. Such cytokine profiling for the inflammation status in tissues, cell cycle analysis in bone marrow, DNA damage assay in lymphocytes, stem cell colony forming assay in bone marrow cells, immune cell populations, kidney health, and tight junctions in intestine, bacterial load in heart, liver, and spleen, and apoptosis in bone marrow and intestine are undergoing in our laboratory.

In summary, RI induced mortality, body-weight loss, and dehydration. Co-therapy of Peg-G-CSF and Ghrelin after RI significantly resulted in enhancement of 30-days survival after RI, mitigation of body-weight loss, hematopoietic acute radiation syndrome and GI acute radiation syndrome in RI mice. These results support the hypothesis/concept of the peg-G-CSF and Ghrelin combination as a co-therapy being efficacious for treating RI. Such a co-therapy could provide timely treatments to RI victims so as to save lives after a nuclear accident.

## Disclaimer

This manuscript has been cleared by the management offices of AFRRI and USU. The views, opinions, and findings within this manuscript are from the authors and do not reflect official policy or positions of the Armed Forces Radiobiology Research Institute, the Uniformed Services University of the Health Sciences, the National Institute of Allergy and Infectious Diseases, the Department of Defense, or the United States government. The authors declare no conflict of interests and no promotion for the products used in the manuscript.

## Data Availability Statement

The original contributions presented in the study are included in the article/Supplementary Material, further inquiries can be directed to the corresponding author.

## Ethics Statement

The animal study was reviewed and approved by the Armed Forces Radiobiology Research Institute IACUC committee. The approved IACUC protocols are 2010-12-016 and 2017-01-002 with a biosample sharing approval.

## Author Contribution

JK conceived and designed the experiment. MZ, BL, JS, MA, and SJ performed the animal experiment. JK, JS, and MA performed biochemical assays. MZ and MA performed histology examination. JGK completed writing the draft of the manuscript. The final manuscript was read, edited, and approved by all authors.

## Funding

This project was supported by NIAID-AFRRI IAA AA112044-001-05000 work plan A and AFRRI RAB310934, RAB33529, and RBB34363. The funding agencies have no roles on the experiments.

## Conflict of Interest

The authors declare that the research was conducted in the absence of any commercial or financial relationships that could be construed as a potential conflict of interest.
